# A High-Quality Genome Sequence of the *Penicillium oxalicum* 5-18 Strain Isolated from a Poplar Plantation Provides Insights into Its Lignocellulose Degradation

**DOI:** 10.3390/ijms241612745

**Published:** 2023-08-13

**Authors:** Shuang Hu, Rui Zhu, Xing-Ye Yu, Bao-Teng Wang, Hong-Hua Ruan, Feng-Jie Jin

**Affiliations:** College of Biology and the Environment, Co-Innovation Center for Sustainable Forestry in Southern China, Nanjing Forestry University, Nanjing 210037, China; shuanghu@njfu.edu.cn (S.H.); zhurui19970706@163.com (R.Z.); yuxy@njfu.edu.cn (X.-Y.Y.); wbt@njfu.edu.cn (B.-T.W.); hhruan@njfu.edu.cn (H.-H.R.)

**Keywords:** *Penicillium oxalicum*, whole genome, xylan, lignocellulose degradation, CAZyme

## Abstract

Studies on the degradation of plant cell wall polysaccharides by fungal extracellular enzymes have attracted recent attention from researchers. Xylan, abundant in hemicellulose, that play great role in connection between cellulose and lignin, has seen interest in its hydrolytic enzymatic complex. In this study, dozens of fungus species spanning genera were isolated from rotting leaves based on their ability to decompose xylan. Among these isolates, a strain with strong xylanase-producing ability was selected for further investigation by genome sequencing. Based on phylogenetic analysis of ITS (rDNA internal transcribed spacer) and LSU (Large subunit 28S rDNA) regions, the isolate was identified as *Penicillium oxalicum*. Morphological analysis also supported this finding. Xylanase activity of this isolated *P. oxalicum* 5-18 strain was recorded to be 30.83 U/mL using the 3,5-dinitro-salicylic acid (DNS) method. Further genome sequencing reveals that sequenced reads were assembled into a 30.78 Mb genome containing 10,074 predicted protein-encoding genes. In total, 439 carbohydrate-active enzymes (CAZymes) encoding genes were predicted, many of which were associated with cellulose, hemicellulose, pectin, chitin and starch degradation. Further analysis and comparison showed that the isolate *P. oxalicum* 5-18 contains a diverse set of CAZyme genes involved in degradation of plant cell wall components, particularly cellulose and hemicellulose. These findings provide us with valuable genetic information about the plant biomass-degrading enzyme system of *P. oxalicum*, facilitating a further exploration of the repertoire of industrially relevant lignocellulolytic enzymes of *P. oxalicum* 5-18.

## 1. Introduction

Conventional energy resources, such as petroleum, gas and coal, which have been widely used in industrial production for many years, are becoming limited and prone to environmental pollution. Plant biomass is considered as the most promising alternative to petrochemicals for the sustainable production of bioenergy due to its abundance, renewability, and low cost [1,2]. Lignocellulose is widely found in plant cell walls, which is primarily composed of three major components (cellulose (40–50%), hemicellulose (25–35%) and lignin (15–20%)) together with other polymers (pectin, protein and wax) in proportion [3]. The composition of lignocellulose also varies by species, tissue position, and age of plants [3]. Due to its recalcitrance, a large number of enzymes are required to degrade lignocellulose fully, the majority of which are included in the carbohydrate-active enzymes (CAZymes) database. These CAZymes are involved in catalyzing the breakdown, biosynthesis, or modification of carbohydrates and glycoconjugates. Based on their catalytic activity and structural similarities, they have been divided into six classes: glycoside hydrolases (GHs), glycosyltransferases (GTs), polysaccharide lyases (PLs), carbohydrate esterases (CEs), enzymes with auxiliary activities (AAs) and carbohydrate-binding modules (CBMs) [4,5].

Hemicellulose, the second most abundant renewable substance in nature, is one of the main constituents of lignocellulose. It serves as a link between lignin and cellulose, improving the toughness of plant cell walls [6,7]. For these reasons, the effective decomposition of hemicellulose is critical to the conversion of lignocellulose into high-value products. In contrast to cellulose, hemicelluloses are heterogeneous polysaccharides comprised of pentoses (D-xylose and D-arabinose), hexoses (D-mannose, D-glucose, and D-galactose), and uronic acids [8]. Thus, the synergistic action of many enzymes is required to thoroughly breakdown hemicellulose.

Xylan, as one of the primary sugar units in plants, accounts for 25–35% of hemicellulose [7]. Due to its importance in hemicellulose, enhancing hydrolysis of xylan by high-efficiency enzymes is beneficial to the utilization of lignocellulose biomass [9]. Therefore, xylanase has emerged as the most important enzyme in the hemicellulose degradation system, which reduces the degree of polymerization of the substrate by cleaving the glycosidic bonds in the xylan backbone [10]. In recent years, it was also subjected to increased attention because of its wide variety of uses in baking, pulp bleaching, and feed industry, among other fields [11]. However, the high cost of enzyme manufacture has also limited its wider adoption. For this reason, research is ongoing to develop a low-cost way to improve enzyme output [12].

Filamentous fungi are excellent decomposers of plant biomass in nature. Species in the phyla Ascomycota and Basidiomycota are able to degrade lignocellulose efficiently due to their diverse CAZymes [13,14]. At present, industrial-scale xylanase production is largely dependent on microorganisms such as species in the genera *Trichoderma* and *Aspergillus* [15,16,17]. However, many species of the genus *Penicillium* were reported to have significant potential for plant biomass degradation and are therefore considered as effective xylanase-producers [18,19,20]. Among these, *Penicillium oxalicum* is regarded as one of the most promising fungi capable of producing a wide range of industrially relevant enzymes [21]. *P. oxalicum* strain 114-2 has been studied for cellulose production in China since 1996 [22,23]. Furthermore, because of its more balanced enzyme system, degradation performance of *P. oxalicum* is generally superior to some other species with comparable functions, such as *Aspergillus niger* and *Trichoderma reesei* [24]. Although fungal xylanases are extensively studied, it is still necessary to screen and develop potential strains, as well as compare their biomass-degrading capacity.

This study aimed to screen high-efficient xylan degrading fungi from poplar plantation leaf litter. After a two-step screening, a strain with strong xylanase production capacity was isolated and identified as *P. oxalicum* based on morphological features and molecular identification. Genome sequencing was performed to obtain further insight into mechanisms involved in biomass degradation of this fungus. Furthermore, the genes encoding CAZymes were annotated in the sequenced genome. Comparative genomic analysis with other species showed that this fungus had more diverse gene-encoding lignocellulose-degrading enzymes.

## 2. Results

### 2.1. Isolation, Morphology and Phylogenetic Analysis of High-Efficient Xylan-Degrading Fungus

**Figure 1:**
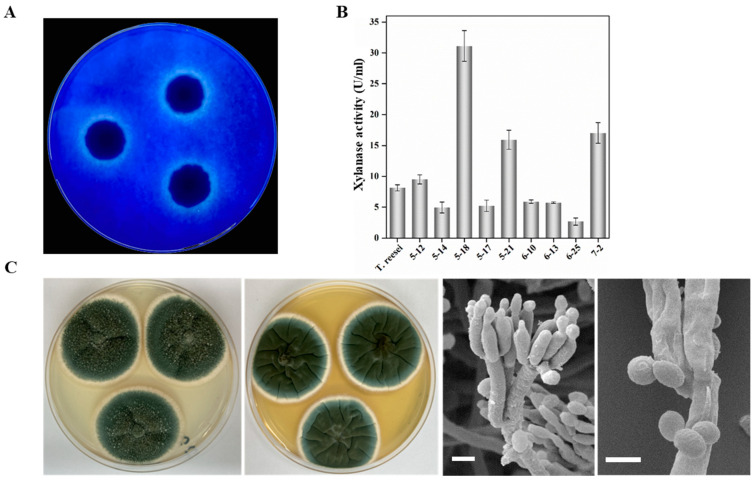
Characteristics of isolate 5-18. (**A**) A clear zone appeared around isolate 5-18 after 3 days of culture on Xylan–Trypan medium at 30 °C. (**B**) Xylanase activity (U/mL) of isolates determined using a DNS method with xylan as the substrate. (**C**) The colonial morphology of isolate 5-18 after 7 days of growth on two media (Left: CYA, Right: YES) at 25 °C, as well as morphological characteristics of conidiophores observed by SEM (Scale bar = 5 μm).

A combination of plate test and enzyme activity assays were used to screen xylan-degrading fungi from litter samples of a poplar plantation. Colonies with clear zones were selected and their xylanase activity was further measured. A total of 10 strains exhibiting notable xylan degradation capacity were isolated from samples. Among these isolates, strain 5-18 was considered to have the strongest xylanase activity with clear transparent circles around the colonies (Figure 1A). Further enzyme assays revealed that strain 5-18 had great potential for xylanase production with activity reaching 30.83 U/mL, substantially higher than that of other isolates and the reference strain *T. reesei* (Figure 1B).

**Figure 2:**
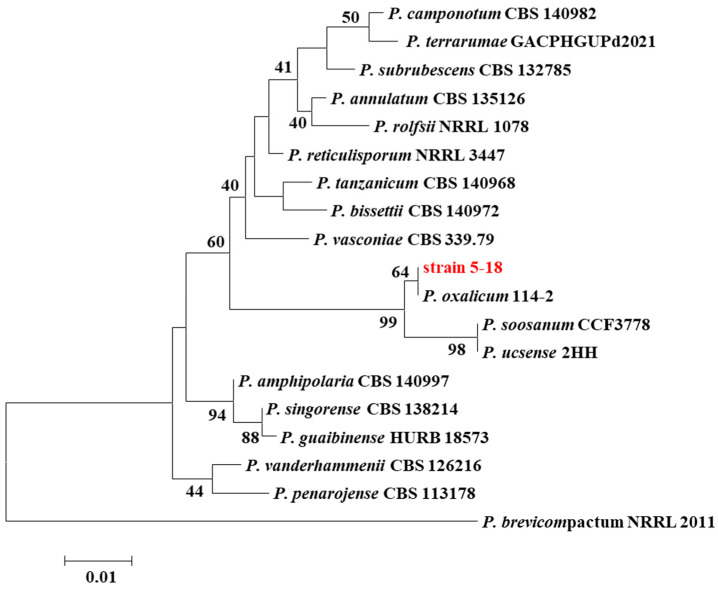
Phylogenetic position of isolate 5-18 inferred from maximum likelihood analysis based on the single ITS region. The bootstraps value (1000 replications) of ML analysis are provided at the nodes. Bars: 0.01 expected nucleotide substitutions per site.

The morphology of strain 5-18 growing on CYA and YES plates was observed after 7 days of incubation at 25 °C in the dark. The colony on CYA was dark green with texture floccose, moderate sporulation, and abundant exudate, while the colony on YES was velvety in texture and dark green fading to blue green in color, with dense spores and no exudate. SEM was used to observe the micromorphology of strain 5-18: conidiophores were strictly biverticillate, stipes slightly rough walled, and had three to six phialides per branch; conidia were broadly ellipsoidal and joined into chains (Figure 1C). These findings suggested that strain 5-18 has the corresponding morphological features of species belonging to genus *Penicillium*. The similarity analysis based on ITS and LSU regions showed that the isolate 5-18 had a higher sequence similarity (100% and 99.8%) with *P. oxalicum* 114-2 for ITS and LSU, respectively. In the phylogenetic tree, based on the ITS gene sequence, strain 5-18 clustered together with *P. oxalicum* 114-2 with high-bootstrap values (Figure 2). In the LSU phylogram, these two strains also form a monophyletic clade distant from other species (Figure S1). Therefore, strain 5-18 was identified as *P. oxalicum* based on morphological and molecular phylogenetic analyses. The strains and related information for molecular phylogenetic analysis in this study are provided in Table S1.

### 2.2. General Genome Features and Functional Annotation

The genome sequencing of strain 5-18 was performed using the Illumina NovaSeq 6000 in conjunction with Nanopore PromethiON 48 platform. An overview of the genome of sequenced strain 5-18 is provided in Table 1. The sequence reads acquired were assembled into nine contigs with a N50 of 3.79 Mb. The final genome size of the assembly was 30.78 Mb, with a guanine and cytosine (G+C) content of 50.55%. Using gene prediction algorithms, a total of 10,074 putative genes were identified with an average coding sequence length of 1454.60 bp. The overall length of the gene was 17,006,221.6 bp, accounting for 55.24% of the whole genome length. With the selected parameters, 10,026 predicted genes could be annotated using blastp (version 2.9.0; E-value < 1 × 10^−5^) and hmmscan (version 3.1; e-value 0.01). Furthermore, non-coding RNA were identified in the genome of strain 5-18 by INFERNAL (version 1.1.2; https://github.com/EddyRivasLab/infernal accessed on 5 August 2023) based on the Rfam database (http://rfam.xfam.org/ accessed on 5 August 2023), including 53 rRNA (5, 2, and 46 copies of 28S, 18S, and 5S rRNAs, respectively), 180 tRNA, 33 snRNAs and 3 sRNA. Other information about the genome, such as exons and introns, was also included in Table 1. A brief comparison of genomic features of strain 5-18 with other lignocellulosic degraders is showed in Table 2. The genome size of strain 5-18 was similar to that of most lignocellulose-degrading fungi, but less than the average genome size of Ascomycota (36.91 Mb) [25]. The G+C content of the genome is in the same way.

Genome features of isolate 5-18 were similar to strain *P. oxalicum* 114-2, as described previously, except that the N50 value of strain 114-2 was lower than that of strain 5-18 (Table 2). This discrepancy may result from recent advances in sequencing technology. In addition, they also differ slightly in genome length and number of protein-coding genes. BUSCO analysis was performed to assess the completeness and integrity of the genome assembly. The results showed that over 98% of the BUSCO genes in the sequenced genome were completed, whereas genes matching the fragmented BUSCOs (F) and the missing sequence (M) accounted for a relatively small fraction, indicating that the genome assembly was reliable and could be used for downstream analysis (Figure S2).

### 2.3. Gene Function Classification

**Figure 3:**
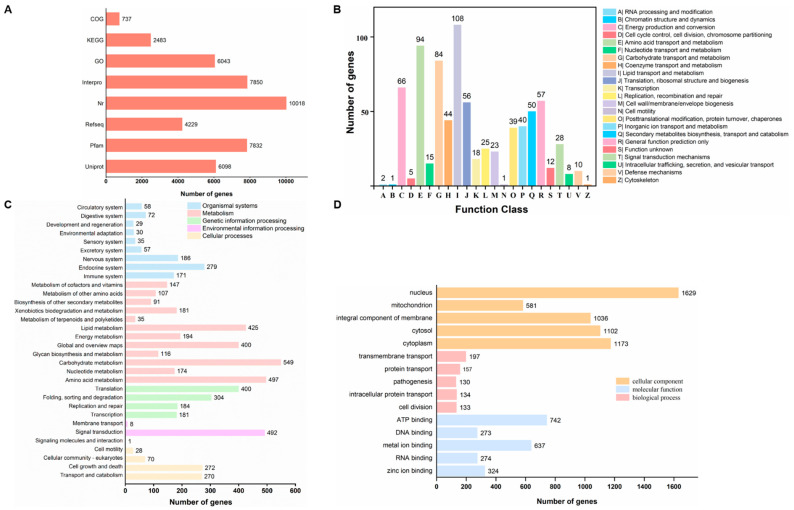
Functional annotations. (**A**) Number of annotated unigenes in *P. oxalicum* 5-18 genome using public databases. (**B**) COG annotation and classification. (**C**) Top 5 terms in each of three KEGG pathway categories. (**D**) Annotation and classification of GO terms.

Functional annotation results indicated that 6098, 7832, 4229, 10,018, 7850, 6043, 2483 and 737 unigenes were successfully annotated in the databases of Uniprot, Pfam, Refseq, Nr, Interpro, GO, KEGG, and COG databases, respectively (Figure 3A).

Gene annotation and functional categorization were conducted using clusters of orthologous groups of proteins (COG), where 737 unigenes closely matched statistics in this database and were assigned into 23 categories (Figure 3B). Among these categories, lipid transport and metabolism (I), amino acid transport and metabolism (E), carbohydrate transport and metabolism (G), energy production and conversion (C) and general function prediction (R) were the most abundant.

KEGG analysis showed that the identified 2483 gene-encoded proteins could be classified into 5 main functional categories and 32 secondary categories (Figure 3C). The metabolism cluster (2752) is the most enriched group, followed by genetic information processing (1060), organismal systems (917), cellular processes (640), and environmental information processing (501). In terms of metabolism, carbohydrate metabolism (549; 22.11%), amino acid metabolism (497; 20.01%) and lipid metabolism (425; 17.12%) were the top three enriched subcategories. The major subcategory in genetic information processing is translation (400; 16.10%), followed by folding, sorting and degradation (304; 12.24%). Signal transduction (492; 19.81%) is the most representative subcategory in environmental information processing. The most enriched subcategories of organismal systems were the endocrine (279; 11.24%) and immune systems (171; 6.89%). For cellular processes, the most dominant pathways were cell growth and death (272; 10.95%) and transport and metabolism (270; 10.87%).

In addition, 59.99% of all genes were successfully annotated to the GO database and divided into three primary functional categories: cellular component, molecular function and biological process. Of cellular components, nucleus (GO:0005634; 26.96%), cytoplasm (GO:0005737; 19.41%), cytosol (GO:0005829; 18.23%) and the integral component of membrane (GO:0016021; 17.14%) were the most enriched. The most common molecular function category was binding, which included ATP binding (GO:0005524; 12.28%), metal ion binding (GO:0046872; 10.54%), zinc ion binding (GO:0008270; 5.36%) and RNA binding (GO:0003723; 4.53%). Transmembrane transport (GO:0055085; 3.26%), protein transport (GO:0015031; 2.6%) and intracellular protein transport (GO:0006886; 2.22%) were the most represented subcategories in biological processes (Figure 3D).

### 2.4. Carbohydrate-Active Enzymes of P. oxalicum 5-18

**Figure 4:**
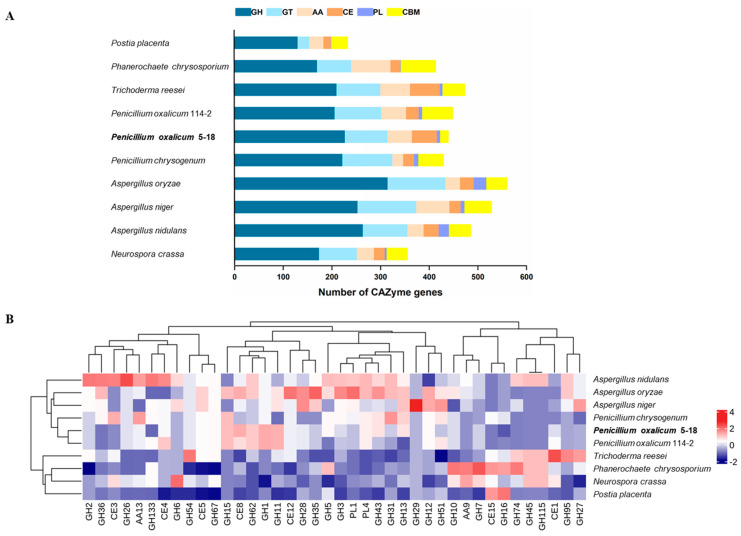
Comparison of CAZyme-encoding genes in *P. oxalicum* 5-18 and other filamentous fungi. (**A**) Comparison of the number of individual CAZyme domains in six CAZyme families of *P. oxalicum* 5-18 and other filamentous fungi. (**B**) Heatmap of the number of CAZyme coding genes in each subfamily associated with plant biomass polymer degradation.

CAZymes, which comprise lignocellulose degradation and other carbohydrate-active enzymes, are classified into six categories in the CAZy database. They can breakdown, modify, or create glycosidic bonds, and play critical roles in the degradation of plant biomass. In view of the importance of CAZymes in biomass utilization, we analyzed the number of various CAZyme-encoding genes in the genome of strain 5-18 and their distribution across individual enzyme families. Meanwhile, several fungal species were chosen for comparison with the CAZyme repertoire of strain 5-18, including well-known biomass degraders from Ascomycetes and Basidiomycetes (Figure 4 and Table S2). A total of 439 putative CAZyme coding genes were found in the genome of *P. oxacilum* 5-18 using dbCAN2 (HMMER algorithm), including 227 glycoside hydrolase genes, 88 glycosyl transferase genes, 7 polysaccharide lyase genes, 51 carbohydrate esterase genes, 50 auxiliary activity genes and 16 carbohydrate-binding module genes. They were classified into 117 distinct subfamilies (60 GH families, 3 PL families, 9 CE families, 8 CBM families, 10 AA families and 27 GT families) (Figure 4A and Table S3A–F). Compared with other biomass degraders selected in this study, strain 5-18 has the intermediate position in the number of CAZyme genes. In addition to three species of *Aspergillus*, strain 5-18 has the most abundant GH family members, with the most abundant subfamilies being GH13, GH3, GH43, GH18 and GH5 (Tables S2 and S3A). The distribution of CAZyme members across the GH families varied greatly among strains. For example, strain 5-18 has three members of the GH154 family, while other species has none, even its relative strain 114-2. In addition, *A. niger* and *Aspergillus oryzae* had approximately double the number of genes in the GH28 family compared with strain 5-18, which were involved in pectin degradation (Table S3A). Strain 5-18 and *T. reesei* have the most CE family members, especially in CE10 where the encoded esterases act on non-carbohydrate substrates. However, no CE10 subfamily members were observed in *P. oxalicum* 114-2 and other species (Table S3D). Except for *Aspergillus nidulans* and *A. oryzae*, none of these species contain more than ten PL genes, whereas strain 5-18 has the same number of PL domains as strain 114-2 (Figure 4A and Table S3E). Furthermore, strain 5-18 has similar AA family distribution with *P. oxalicum* 114-2 and *T. reesei*, and the AA7 subfamily is the most abundant, mainly acting on gluco- or chito-oligosaccharide oxidases and cellooligosaccharide dehydrogenization (Table S3C). Unfortunately, the number of CBMs in strain 5-18 is substantially lower than in other species, particularly *P. oxalicum* 114-2 (Figure 4A and Table S2). The putative CBM family members in *P. oxalicum* 114-2 are mainly distributed in 1, 18, and 50 subfamilies, which contributed to cellulose- and chitin-binding, while none of these subfamily members were predicted in the genome of strain 5-18 (Table S3F).

To evaluate the ability to degrade plant cell walls, we compared plant cell wall degrading-enzyme (PCWDE) genes in various CAZyme subfamilies of strain 5-18 and nine other lignocellulose-degrading fungi (Figure 4B, details in Table S4). The synergistic action of multiple enzymes is essential for the utilization of biomass. For instance, degradation of cellulose requires endoglucanases (EG), cellobiohydrolases (CBH), and β-glucosidases (BGL). These enzymes belong primarily to seven CAZyme families in CAZy database, including GH1, GH3, GH5, GH6, GH7, GH12 and GH45. In the genome of strain 5-18, putative candidates for above three cellulase types span six distinct subfamilies and the number of GH5 and GH13 family members is higher in strain 5-18 than in *P. oxalicum* 114-2. Although *T. reesei* is known as the type strain for cellulase production, a fewer number of genes related to hemicellulase were found in the *T. reesei* genome, such as endo-1,4-β-xylanase, 1,4-β-xylosidase and endo-1,4-β-mannosidase. The endo-1,4-β-xylanases in CAZyme database mainly belong to GH10 and GH11, while others are also catalogued to GH5-6, GH8, GH26, GH30, GH43 and GH98. 1,4-β-xylosidases are classified into GH3, GH30, GH39, GH43, GH52 and GH54. In the genome of strain 5-18, the annotated gene-encoding enzymes belonging to GH10, 11, 3, 51, 54 and 62 were more abundant than those of *T. reesei*. These findings are consistent with previous reports that *T. reesei* is inferior to *Penicillium* spp. in terms of degrading hemicellulose [21,31]. Compared with *P. oxalicum* 114-2, a large number of protein-coding genes containing GH13 and GH31 domains were found in strain 5-18 (Figure 4B). Both CAZyme subfamilies have starch-degrading activity, such as α-glucosidase and α-amylase. This finding suggests that strain 5-18 has a potentially stronger ability to degrade starch. Apart from 11 gene-encoded proteins containing GH 28 domain, pectin/pectate lyase (PL1), rhamnogalacturonan lyase (PL4) and several pectinesterase (CE8 and CE12), genes were also found for pectin degradation in the genome of strain 5-18.

#### 2.4.1. Degradation of Cellulose

**Figure 5:**
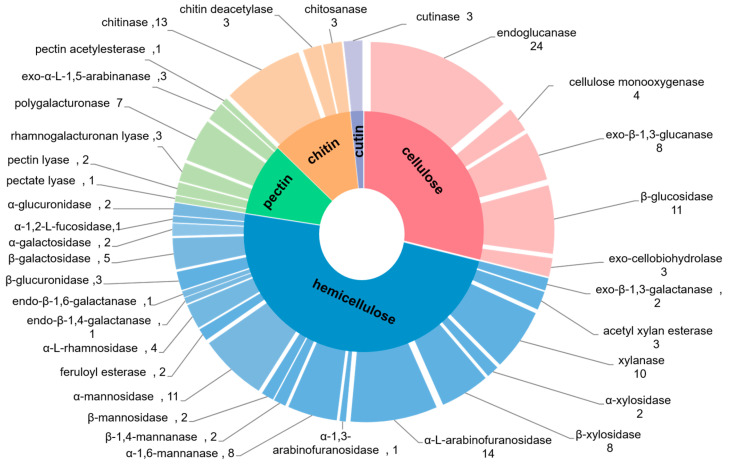
Classification of plant cell wall degrading enzymes in strain 5-18.

The diversity and strain specificity of the PCWDE genes found in the *P. oxalicum* 5-18 genome revealed the importance of this strain in the degradation and utilization of complex carbohydrates as nutrients. Further analysis showed that 181 enzymes were annotated as being linked with degradation of lignocellulose, including 50 cellulases, 84 hemicellulases, 17 enzymes engaged in pectin decomposition, and others (Figure 5 and Table S5).

Cellulose mainly exists in plant cell walls, and is a highly stable linear homopolymer with high-molecular weight. It consists of many glucose units connected by β-1,4 linkages and is resistant to enzyme hydrolysis due to the formation of insoluble crystalline microfibrils [32,33,34]. Therefore, the synergistic action of multiple enzymes is required to completely degrade cellulose. A variety of enzymes related to cellulose degradation were detected in the genome of *P. oxalicum* 5-18, mainly including 32 glucanases, 11 β –glucosidase (BGLs) and 3 cellobiohydrolases (CBHs), among others (Figure 5). Earlier studies have shown that EGs hydrolyze cellulose at random locations in less crystalline regions, followed by CBHs acting on the non-reducing ends of the cellulose polymer. Finally, the released cellobiose is further cleaved by BGLs to produce glucose [35]. In addition, four AA9 genes (g3006, g3546, g5364 and g8034) encoding lytic polysaccharide monooxygenases (LPMOs) were also identified in *P. oxalicum* 5-18 genome, as ortholog of cel61a from *P. oxalicum* 114-2 (Figure 5 and Table S5). Research shows that LPMOs are beneficial to the destruction of cellulose by catalyzing the initial oxidative cleavage of recalcitrant cellulose [36]. Therefore, these findings suggest that strain 5-18 has great potential for cellulose degradation.

#### 2.4.2. Degradation of Hemicellulose

Further investigation of the CAZyme family indicated that *P. oxalicum* 5-18 seemed to have superior capacity to degrade hemicellulose. As the major component of hemicellulose, hydrolysis of xylan is a key step in utilizing abundant lignocellulosic biomass. Xylans feature a linear backbone made up of β-1,4-linked D-xylopyranose residues that may be substituted for branches carrying other residues, such as acetyl, arabinosyl, D-galactose and glucuronosyl, which vary depending on plant species and extraction techniques [7,37]. In this study, seven kinds of enzymes involved in xylan degradation were predicted: xylanase, xylosidase, glucuronidase, α-L-arabinofuranosidase, galactosidase, acetyl xylan esterase and feruloyl esterase (Figure 5). Among these, 10 xylanases and 10 xylosidases collaborate to cleave the main chain of xylan. Xylanase acts on glycosidic bonds in the xylan backbone to destroy the degree of polymerization of the substrate, and the xylobiose generated by xylanase is further degraded by β-xylosidases [10,38]. Glucuronidase and α-L-arabinofuranosidase are the enzymes necessary for completely xylan conversion, which can eliminate branched substitutions. Previous studies have found that glucuronidase may remove the 4-*O*-methyl D-glucuronic acid substituents from xylan branches [39]. α-L-arabinofuranosidase genes were also found in sufficient abundance in the strain 5-18 genome, indicating that strain 5-18 has a great capacity to hydrolyze compounds containing arabinofuranoside bonds by removing arabinose side groups attached to xylooligosaccharides generated by xylanases [40].

Seven genes encoding galactosidases, which function to remove galactopyranosyl residues from xyloglucan oligosaccharides, were identified in the genome of strain 5-18 [41]. Five genes encoding three acetyl xylan esterases and two feruloyl esterases were also found in the strain 5-18 genome. The main function of these carbohydrate esterases (CEs) is to remove the side substitutes from the xylan backbone, exposing the xylan backbone to xylanase directly, which is conducive to the overall deconstruction process [42]. As a result, these enzymes, mentioned above, completely breakdown xylan through synergistic interactions. In addition, four genes encoding α-L-rhamnosidases (GH78), as an auxiliary enzyme that contributes to hemicellulose degradation [43], were also found in the genome of strain 5-18.

#### 2.4.3. Degradation of Other Plant Cell Wall Polymers

In addition to genes involved in cellulose and hemicellulose degradation, numerous gene-encoding enzymes responsible to other plant cell wall polymer degradation were also discovered in strain 5-18, such as pectin, chitin and cutin (Figure 5 and Table S5). We found that strain 5-18 contained a larger number of genes required for degradation of xyloglucan and starch, although the amylo-α-1,6-glucosidase genes were missing from this genome. A detailed analysis also found an abundance of pectinase genes in the genome of strain 5-18 (Figure 5), including seven polygalacturonases, at least three pectin/pectate lyases, three rhamnogalacturonan lyases, and one pectin acetylesterase, among others. Of these, polygalacturonase and pectin/pectate lyase are members of the GH28 and PL1 families, respectively. Polygalacturonase primarily catalyzes the hydrolytic cleavage of the polygalacturonic acid chain in the presence of water by separating the α-1,4-glycosidic bonds between galacturonic monomers [44]; pectin/pectate lyase and rhamnogalacturonan lyase cleave α-1,4-glycosidic bonds to liberate oligosaccharides with 4-deoxy-α-d-mann-4-enuronosyl groups [45,46]; and pectin acetylesterase hydrolyzes acetylester bonds [47]. Compared to other investigated Ascomycota fungi, *N. crassa* and *T. reesei* have fewer pectinase genes, and no pectin/pectate and rhamnogalacturonan lyase genes were found in *T. reesei*, as was also reported in *P. chrysosporium* and *P. placenta* (Table S3). Furthermore, 19 chitin-degrading enzymes were identified in the strain 5-18 genome, largely belonging to three CAZyme subfamilies (GH18, GH75 and CE4), including 13 chitinases, 3 chitin deacetylases and 3 chitosanases (Figure 5). In addition, α-L-arabinofuranosidase and galactosidase, which are engaged in hemicellulose degradation, are also implicated in pectin breakdown [48].

## 3. Discussion

Filamentous fungi play a significant role in the degradation of lignocellulose in nature, and fungal CAZymes have been widely applied in industrial processes to convert plant biomass into valued-added products. Brown and white rot fungi are the most common biomass-degrading fungi on earth, belonging to the phyla Basidiomycota and Ascomycota, respectively [49]. Generally, CAZymes are more abundant in Ascomycota species than in Basidiomycota, which may be attributed to their long evolutionary history and capacity to exploit a diverse spectrum of organic substances, such as cellulose, lignin, and other biopolymers [50]. Among the Ascomycetes, *Penicillium* is well-known genus, distributed throughout the world, and its species serve important roles in the breakdown of organic matter, with many of them serving as enzyme factories [51]. Higher quality and accuracy of genetic information of species can help reveal the mechanisms of lignocellulose degradation, and contribute to genetic manipulation of many industrial microbial strains to reduce enzyme-production cost and improve efficiency. In recent years, Illumina and PacBio sequencing technologies were widely used for genome assembling due to their high efficiency [52]. In this study, we aimed to systematically explore the lignocellulolytic potential of the isolated fungus 5-18 using genome sequencing analysis. This strain was obtained from the litter of a poplar plantation. Phylogenetic analysis of ITS and LSU regions, as well as further morphological features, showed that strain 5-18 was most closely related to *P. oxalicum* 114-2. Interestingly, strain 114-2 was also isolated from decayed straw-covered soil in 1979 [23], demonstrating that this species is extensively distributed in the environment and contributes to the degradation of lignocellulose.

Based on the merit and demerit of different sequencing technologies, we adopted an integrated strategy of sequence assembly, that is, the data generated by the third-generation sequencing was used for genome sequence assembly, and the second-generation sequencing data was used for error correction. Genome sequencing results showed that the total genome size of this strain was 30.78 Mb, which is within the range of common plant biomass-degrading fungi, such as *Aspergillus* spp., *Trichoderma* spp., and *Neurospora* spp, especially close to its relative strain 114-2 (30.17 Mbp) [53]. Gene annotation was conducted through a series of various databases in order to precisely evaluate the genome characteristics of strain 5-18 from different aspects. Based on Nr annotation, 97.41% of protein-coding genes in the isolate 5-18 genome were homogenous to *P. oxalicum* strain 114-2, which also confirmed slight differences between the two strains. Due to the complexity of the structure and composition of natural lignocellulosic materials, a variety of enzymes with substrate specificity are required for complete hydrolysis [54]. In this study, we focused more attention on the gene-encoding CAZymes, which is involved in lignocellulose degradation. Results revealed that strain 5-18 included a large number of CAZyme coding genes, distributed in at least 117 distinct families, many of which were involved in degradation of cellulose, xylan, galactomannan, xyloglucan, pectin and starch. This finding shows that this fungus may be an excellent producer of extracellular enzymes that degrade plant biomass.

*P. oxalicum* 114-2 was used for industrial-scale manufacture of lignocellulolytic enzymes for more than 20 years [21]. Compared with *T. reesei*, a fungus widely used for production of cellulase, *P. oxalicum* 114-2 possesses a more balanced enzyme system and stronger xylanase activity [21]. In this study, we found that strain 5-18 had slight differences in the distribution and amount of CAZyme genes compared with the strain *P. oxalicum* 114-2. As reported, cellulose-binding CBM1 is particularly enriched in Ascomycetes, with an average of 30% CBMs belonging to CBM 1 family [55], which contributed to the attachment to various substrate surfaces for higher degradation efficiency [21]. Comparative genomic analysis showed that *P. oxalicum* 114-2 contained a large number of gene-encoding CBM1-containing proteins, while strain 5-18 had none. However, this may not be a disadvantage as studies have shown that in presence of a low water content substrate, the enzyme exhibits the same hydrolysis effect regardless of whether CBM protein is present [55,56]. Therefore, the lower number of CBM protein-coding genes in the strain 5-18 genome could be attributed to their dry habitat (dry litter). Additionally, strain 5-18 also has more abundant genes belonging to the GH and CE class, especially the CE 10 subfamily, suggesting that this fungus has genetic potential to utilize non-carbohydrate. These findings are consistent with earlier studies hypothesizing that two distinct strains of the same species may also differ in terms of biomass hydrolysis and enzyme secretion [57]. This phenomenon is largely due to post-genomic differences between strains [58].

Although strain 5-18 was not studied as intensively as strain 114-2, the results of the comparative genomic analysis suggested that this strain possess numerous enzymes for lignocellulose degradation, particularly xylan in hemicellulose. As various CAZymes have synergistic or complementary effects on the same substrate, it is considered that the diversity and balance of enzyme systems are more important for lignocellulose degradation [59,60]. Our research confirmed that strain 5-18 could be used as an excellent lignocellulose-degrading enzyme producer in the future. In addition, this fungus has advantages in other aspects due to the functional complexity of the numerous enzymes identified. Strain 5-18 possess 8 exo-β-1,3-glucanases in GH5 and GH55 families, and in addition to cellulose degradation, they are also involved in morphogenetic–morphologic processes, mobilization of β-glucan and fungal interspecies competition [61,62]. The genomic data could reveal degradation potential of fungi, but the gene expression of various species is often induced by different carbon source at the transcriptomic level actually [24,63]. For example, *Trametes hirsuta* expressed exo-β-1,3-glucanase in presence of glucose, but no exo-β-1,3-glucanase was found in oat straw media. The function of this enzyme may be substituted by other glycosidases induced by oat straw [64]. Therefore, the study of transcriptome and proteomic-level has further practical significance for revealing actual expression level of specific proteins. In summary, the accurate genomic information of strain 5-18 reveals its potential to degrade lignocellulose, which lays a foundation for further genetic engineering investigation.

## 4. Materials and Methods

### 4.1. Isolation and Screening of Xylan-Degrading Fungi

Rotting leaf litter samples were collected from a poplar plantation (32°51′42″ N, 120°48′2″ E) located in Dongtai, Jiangsu Province, China. Approximately 0.5 g leaf samples were cut into small pieces with sterile scissors, placed in a 50 mL sterilized centrifugal tube with 10 mL of sterile water, and shaken well for one minute. This mixture was subjected to a series of gradient dilutions (10^−2^, 10^−3^, 10^−4^, 10^−5^). A total of 300 µL aliquots of each serial dilution were spread onto the agar plate with xylan as the sole carbon source, and 50 mg/L streptomycin was added to inhibit bacterial growth. The agar plates were then incubated in the dark at 30 °C for 3–10 days. The screening medium was composed of xylan (JianglaiBio, Shanghai, China) 10 g/L, 4 g/L (NH_4_)_2_SO_4_, 1 g/L peptone, 0.5 g/L MgSO_4_·7H_2_O, 1 g/L KH_2_PO_4_, 0.04 g/L Trypan blue (Sigma-aldrich, St. Louis, MO, USA) and 20 g/L agar.

Strains that formed clear zones surrounding colonies were chosen and purified using repeated plate streaking method for additional enzyme assay. The xylanase activity of the isolates was examined by measuring the amount of reducing sugar that was released from the substrate using the 3,5-dinitro-salicylic acid (DNS) method [65]. The reaction mixture consists of 0.5 mL of a suitably diluted enzyme suspended with 1.5 mL 1% (*w*/*v*) xylan in an acetate buffer (pH 5.5). After incubation at 50 °C for 10 min, 3 mL DNS was added to halt the reaction, then the mixture was boiled for 5 min. The resulting mixture was diluted appropriately, and the presence of reducing sugar was determined using an ultraviolet spectrophotometer at 540 nm. All experiments were carried out in triplicate. One unit (U) of xylanase activity is defined as the quantity of enzyme required to release 1 μmol of reducing sugar per minute under assay conditions. For long-term preservation of the cultures, all strains were stored in 20% glycerol at −80 °C for further analysis.

### 4.2. Morphological Characteristics of the Isolates

The morphological traits of newly isolated strains were initially investigated using conventional methods that included macroscopic and microscopic aspects. Macroscopic characteristics, such as appearance, texture, soluble pigment and other important features, were studied after 7 days of incubation on CYA (Czapek yeast autolysate agar, Kanglang, Shanghai, China) and YES (yeast extract agar) media under standard incubation conditions [66]. All incubation was finished by inoculating the spore suspension (1 μL per spot) at three points to obtain the pure single colony. A highly flexible scanning electron microscope (SEM) Prisma E (Thermo Fisher Scientific Company, Waltham, MA, USA) was used for microscopic morphology observation and image analysis. Colonies, cultivated on MEA (malt extract agar, Solarbio, Beijing, China) medium for 7 days, were used to prepare slides for microscopic observation.

### 4.3. Molecular Identification and Phylogenetic Analyses

The molecular identification of isolate 5-18 was performed based on the sequence analysis of polymerase chain reaction (PCR) amplification in a GeneAmP 9700 system (Applied Biosystems, Foster City, CA, USA). The PCR enzyme 2 × T5 Direct PCR kit (TsingKe; Beijing, China) was used to amplify the targeted genes from 2–3 day old mycelia grown on Potato dextrose agar (PDA; BD Difco, Sparks, MD, USA). The ITS (internal transcribe spacer) and LSU (Large subunit 28S rDNA) regions were amplified using primers ITS4/ITS5 [67] and NL1/NL4 [68], respectively.

The PCR products were visualized using 0.8% agarose gel electrophoresis and the acquired DNA sequence data were compared to available sequences in GenBank using the Blast-n program on NCBI (National Center for Biotechnology Information). Sequences were aligned using ClustalX [69], then manually optimized using BioEdit [70]. The ITS, LSU regions of strain 5-18 and other relevant strains were selected to conduct phylogenetic trees based on a single gene (ITS or LSU). The individual gene data sets were used to generate the respective Maximum-Likelihood (ML) trees using the software MEGA7.0 [71] and the Kimura two-parameter model. The p-distance substitution model with 1000 bootstrap replications was used for reliability assessment of each clade.

### 4.4. Genome Sequencing, Assembly, Gene Prediction, and Annotation

The high-quality genomic DNA was extracted from mycelia using the CTAB method from the pure cultures, which was sequenced using a combination of the Illumina NovaSeq 6000 (Illumina Inc., San Diego, CA, USA) and Oxford Nanopore PromethiON 48 (Oxford Nanopore Technologies, Oxford, UK) platform. For second-generation sequencing technology on the novaseq 6000 platform, qualified DNA was fragmented randomly using the Covaris ultrasonic system. The paired-end fragment libraries were sequenced according to the Illumina NovaSeq 6000 system’s protocol. After filtration, 4.3 Gb of clean data was retrieved. For third generation sequencing on Nanopore platforms, the genomic DNA was sheared using ultrasonics and the library was constructed according to Oxford nanopore technology sequencing instruction manual. The Nanopore sequencing yielded 4.4 Gb of clean data in 856,742 quality-filtered reads from Flow cells. All subsequent analyses were based on the high-quality clean data. The resulting reads were assembled using NECAT v20200119 (https://github.com/xiaochuanle/NECAT accessed on 5 August 2023) and nextDenovo v2.3.1 (https://github.com/Nextomics/NextDenovo accessed on 5 August 2023).

Racon v1.4.13 (https://github.com/isovic/racon accessed on 5 August 2023) and Purge Haplotigs (https://bitbucket.org/mroachawri/purge_haplotigs accessed on 5 August 2023) were used for sequence correction. Genomescope v1.0.0 software (https://github.com/schatzlab/genomescope accessed on 5 August 2023) was used to assess the size of genome. Genome completeness was evaluated using Bench marking Universal Single-Copy Orthologs v4.1.4 (BUSCO) [72], whereas braker v2.1.5, Augustus v2.6.1 and GeneMark-EX 4.33 were employed for further gene prediction. Using the BLAST algorithm with default parameters, the predicted genes were functionally annotated using homologous alignment against the GO (Gene Ontology), KEGG (Kyoto Encyclopedia of Genes and Genomes), UniProt, COG (Cluster of Orthologous Groups) and NCBI NR databases. CAZyme domain-containing genes were discovered and predicted using the HMMER 3.2.1 package (http://hmmer.org/ accessed on 5 August 2023) in conjunction with the dbCAN CAZyme database (http://www.cazy.org/ accessed on 5 August 2023) [73].

## 5. Conclusions

In this study, a strain with strong xylan-degrading ability was isolated and identified as *P. oxalicum* based on molecular phylogenetic and morphology analyses. A 30.78 Mb full-length genome containing 10,074 protein-coding genes was obtained using genome sequencing and assembly. Further gene annotation analysis showed that the isolate *P. oxalicum* 5-18 contains a diverse set of CAZyme genes involved in degradation of plant cell wall components. Compared with *P. oxalicum* 114-2, *P. oxalicum* 5-18 includes more GH and CE family members, which are mainly engaged in cellulose and hemicellulose degradation, suggesting that the fungus has the theoretical potential to breakdown and use plant biomass. Furthermore, KEGG and COG analyses also revealed the main biological function pathways of this fungus. Data obtained, along with further comparative genomic analysis, have expanded our in-depth understanding of the function of *P. oxalicum*, in particular, the degradation mechanisms of lignocellulose, and providing valuable insights into fundamental biology and industrial biotechnology.

## Figures and Tables

**Table 1:** Overview of *P. oxalicum* 5-18 genome sequenced in this study.

Genome Assembly	Value
Total length (bp)	30,780,966
Number of contigs	9
Max (bp)	5,859,503
Min (bp)	51,307
Median (bp)	3,697,257
N50 (bp)	3,787,046
G+C content (%)	50.55
BUSCO (%)	98.8
**Protein annotation**	
Total number of predicted protein-encoding genes	10,074
Proteins/genes with functional annotation	10,026
**Non-coding RNAs**	
tRNAs	180
rRNAs	53
snRNAs	33
sRNA	3
**Exon**	
Number of exon	31,080
Total exon length (Mb)	15
Average of exon length (bp)	471.48
Average of exon number	3.09
**Intron**	
Number of intron	21,006
Total intron length (bp)	2,151,025
Average of intron length (bp)	102.4

**Table 2:** Genome features of strain 5-18 and other lignocellulose-degrading fungi.

Organisms	Phylum	Scaffold	N50 (bp)	Genome Size (Mb)	G + C (%)	Number of Protein Coding Genes	References
*Penicillium oxalicum* 5-18	Ascomycota	-	3,787,046	30.78	50.55	10,074	In this study
*Penicillium oxalicum* 114-2		9	157,054	30.17	50.60	10,013	[26]
*Penicillium chrysogenum*		49	-	32.22	49.00	13,911	[27]
*Neurospora crassa*		21	656,009	41.1	48.23	10,812	[27]
*Aspergillus nidulans*		8	679,860	29.83	50.37	10,455	[27]
*Aspergillus niger*		-	-	33.98	50.40	10,785	[27]
*Aspergillus oryzae*		12	2,324,132	37.91	48.26	12,074	[28]
*Trichoderma reesei*		89	-	34.1	51.00	9143	[29]
*Phanerochaete chrysosporium*	Basidiomycota	1323	78,962	29.9	57.00	11,777	[27]
*Postia placenta*		1842	73,576	36.37	53.60	11,246	[30]

## Data Availability

The data presented in the study has been deposited at DDBJ/ENA/GenBank under the accession JASKYK000000000.

## Supplementary Materials

The supporting information can be downloaded at: https://www.mdpi.com/article/10.3390/ijms241612745/s1.
