# Supplementary material for: A High-Quality Genome Sequence of the Penicillium oxalicum 5-18 Strain Isolated from a Poplar Plantation Provides Insights into Its Lignocellulose Degradation
Source: Int J Mol Sci. 2023 Aug 13;24(16):12745. doi: 10.3390/ijms241612745 (PMC10454814; doi:10.3390/ijms241612745)
Supplement: Supplementary file 1 [file ijms-24-12745-s001.zip › Figures.pdf]

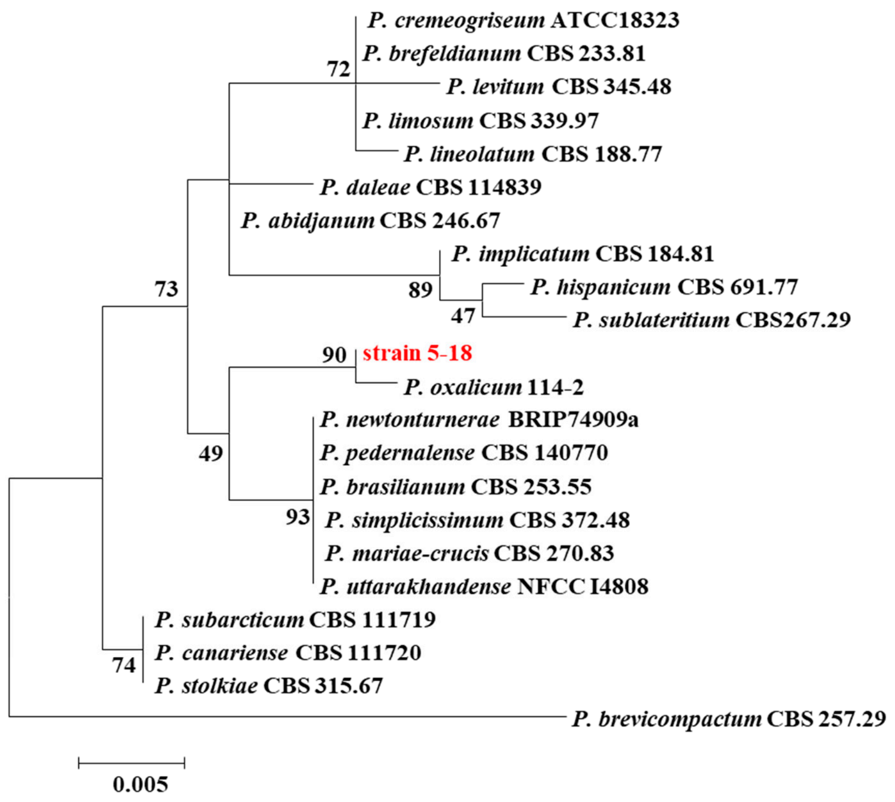

Figure S1 Phylogenetic position of *P. oxalicum* 5-18 inferred from maximum likelihood analysis based on the single LSU region. Sequence accession numbers used for phylogenetic analysis in this study were shown before species name. The bootstraps value (1000 replications) of ML analysis are given at the nodes. Bars: 0.005 expected nucleotide substitutions per site.

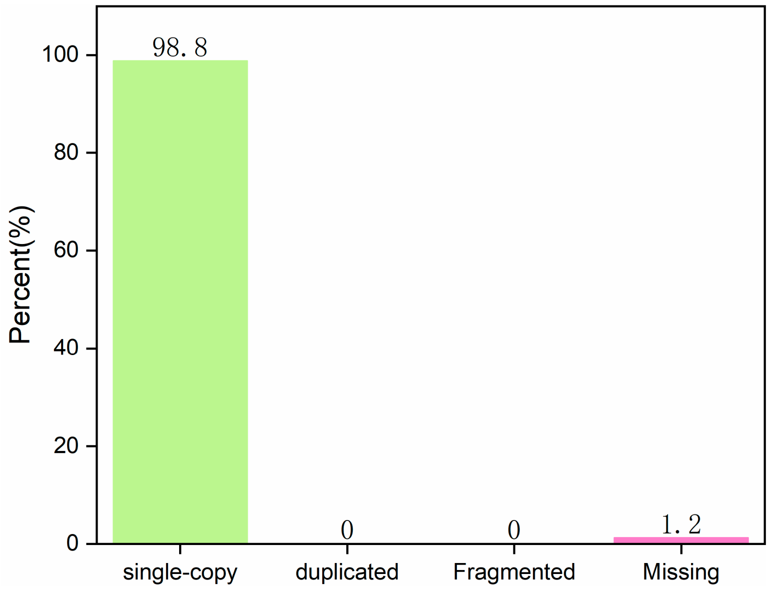

Figure S2 BUSCO analysis of genome.
